# Surgical Antimicrobial Prophylaxis in Abdominal Surgery for Neonates and Paediatrics: A RAND/UCLA Appropriateness Method Consensus Study

**DOI:** 10.3390/antibiotics11020279

**Published:** 2022-02-21

**Authors:** Sonia Bianchini, Erika Rigotti, Sara Monaco, Laura Nicoletti, Cinzia Auriti, Elio Castagnola, Giorgio Conti, Luisa Galli, Mario Giuffrè, Stefania La Grutta, Laura Lancella, Andrea Lo Vecchio, Giuseppe Maglietta, Nicola Petrosillo, Carlo Pietrasanta, Nicola Principi, Simonetta Tesoro, Elisabetta Venturini, Giorgio Piacentini, Mario Lima, Annamaria Staiano, Susanna Esposito, the Peri-Operative Prophylaxis in Neonatal and Paediatric Age (POP-NeoPed) Study Group

**Affiliations:** 1 Pediatric Clinic, University Hospital, Department of Medicine and Surgery, University of Parma, 43126 Parma, Italy; bianchini.sonia@outlook.it (S.B.); s.monaco1410@gmail.com (S.M.); laura.nicoletti@studenti.unipr.it (L.N.); 2 Pediatric Unit, Department of Surgical Sciences, Dentistry, Gynecology and Pediatrics, University of Verona, 37124 Verona, Italy; erika.rigotti@aovr.veneto.it (E.R.); giorgio.piacentini@univr.it (G.P.); 3 Neonatology and Neonatal Intensive Care Unit, IRCCS Bambino Gesù Children’s Hospital, 00165 Rome, Italy; cinzia.auriti@opbg.net; 4 Infectious Diseases Unit, IRCCS Giannina Gaslini, 16147 Genoa, Italy; eliocastagnola@gaslini.org; 5 Pediatric ICU and Trauma Center, Fondazione Policlinico Universitario A. Gemelli IRCCS, 00165 Rome, Italy; giorgio.conti@unicatt.it; 6 Pediatric Infectious Disease Unit, Meyer Children’s Hospital, 50139 Florence, Italy; luisa.galli@unifi.it (L.G.); elisabetta.venturini@meyer.it (E.V.); 7 Department of Health Promotion, Mother and Child Care, Internal Medicine and Medical Specialties “G. D’Alessandro”, University of Palermo, 90141 Palermo, Italy; mario.giuffre@unipa.it; 8 Institute for Biomedical Research and Innovation, National Research Council, 90146 Palermo, Italy; stefania.lagrutta@irib.cnr.it; 9 Paediatric and Infectious Disease Unit, Academic Department of Pediatrics, IRCCS Bambino Gesù Children’s Hospital, 00165 Rome, Italy; laura.lancella@opbg.net; 10 Department of Translational Medical Science, Section of Pediatrics, University of Naples “Federico II”, Via D. Montesano 49, 80131 Naples, Italy; andrealovecchio@gmail.com (A.L.V.); staiano@unina.it (A.S.); 11 Research and Innovation Unit, University Hospital of Parma, 43126 Parma, Italy; gmaglietta@ao.pr.it; 12 UniCampus University, 00125 Rome, Italy; n.petrosillo@unicampus.it; 13 Fondazione IRCCS Ca’ Granda Ospedale Maggiore Policlinico, Department of Mother, Child and Infant, NICU, 20122 Milan, Italy; carlo.pietrasanta@unimi.it; 14 Università degli Studi di Milano, 20122 Milan, Italy; nicola.principi@unimi.it; 15 Division of Anesthesia, Analgesia and Intensive Care, Department of Surgical and Biomedical Sciences, University of Perugia, 06129 Perugia, Italy; simonettatesoro@gmail.com; 16 Paediatric Surgery, IRCCS Azienda Ospedaliera-Universitaria di Bologna, 40138 Bologna, Italy; mario.lima@unibo.it

**Keywords:** abdominal surgery, appendectomy, gastrointestinal endoscopy, liver surgery, pancreas surgery

## Abstract

Surgical site infections (SSIs), i.e., surgery-related infections that occur within 30 days after surgery without an implant and within one year if an implant is placed, complicate surgical procedures in up to 10% of cases, but an underestimation of the data is possible since about 50% of SSIs occur after the hospital discharge. Gastrointestinal surgical procedures are among the surgical procedures with the highest risk of SSIs, especially when colon surgery is considered. Data that were collected from children seem to indicate that the risk of SSIs can be higher than in adults. This consensus document describes the use of preoperative antibiotic prophylaxis in neonates and children that are undergoing abdominal surgery and has the purpose of providing guidance to healthcare professionals who take care of children to avoid unnecessary and dangerous use of antibiotics in these patients. The following surgical procedures were analyzed: (1) gastrointestinal endoscopy; (2) abdominal surgery with a laparoscopic or laparotomy approach; (3) small bowel surgery; (4) appendectomy; (5) abdominal wall defect correction interventions; (6) ileo-colic perforation; (7) colorectal procedures; (8) biliary tract procedures; and (9) surgery on the liver or pancreas. Thanks to the multidisciplinary contribution of experts belonging to the most important Italian scientific societies that take care of neonates and children, this document presents an invaluable reference tool for perioperative antibiotic prophylaxis in the paediatric and neonatal populations.

## 1. Introduction

Surgical site infections (SSIs), i.e., surgery-related infections that occur within 30 days after surgery without an implant and within one year if an implant is placed, complicate surgical procedures in up to 10% of cases, but an underestimation of the data is possible since about 50% of SSIs occurs after the hospital discharge [1,2]. They are classified as incisional or organ/space infections according to whether they concern the incision site or the organ(s) and/or spaces that were manipulated during surgery. SSIs are the most common and costliest hospital-acquired infections, as they account for approximately 20% of all hospital-acquired infections and place a substantial burden on health systems and service payers [3]. Although data are not available in the paediatric population, it has been shown that adult patients with SSIs have a longer duration of hospitalization and, after discharge, frequently need emergency department visits, hospital readmission and, in rare cases, reoperation [3]. Moreover, they have a 2–11 times greater risk of death than patients without SSI. Finally, the mean healthcare costs for a patient with SSI are approximately twice the costs for a patient without SSI [1,2,3].

As bacteria play a major role in SSIs [4,5,6,7,8,9,10], since the beginning of the antibiotic era, patients that are undergoing surgery have been prophylactically-treated with antibiotics. Unfortunately, antibiotics were frequently applied without considering which type of surgery and patients could really benefit from prophylaxis, which antibiotics could be effective in SSIs prevention, and what was the most appropriate drug dosage and duration of administration [10,11,12]. When data in this regard were collected, it was clearly seen that antibiotic abuse and misuse increased drug-related adverse events, healthcare costs, and the emergence of drug-resistant organisms [6,7,8,9]. 

To contain these problems and rationalize the use of antibiotic prophylaxis, several scientific institutions have prepared and occasionally updated guidelines for SSI prevention [10,11,12,13]. Attempts to identify which surgical patients were more prone to SSIs, which pathogens were more frequently associated with SSIs, which antibiotics could be more effective, and which were the most appropriate prescriptive modalities were made [10,11,12,13]. Despite this, definitive recommendations could be made for only some surgeries, as for several of them, randomized, double-blind, placebo-controlled studies comparing patients that were receiving different antibiotic options to placebo or no prophylaxis were not available [14]. This is even more evident in children, a group of patients who, especially in the first years of life, are at the highest risk of infections. Studies in children are scarce, and the use of antibiotic prophylaxis among children is exposed to risks of mistakes, even more so than in adults [14].

Gastrointestinal surgical procedures are among the surgical procedures with the highest risk of SSIs, especially when colon surgery is considered. A surveillance of SSIs in Europe during 2010–2011 showed that, among the most common surgical procedures, the incidence of SSI was 9.5% in colon surgery compared to 3.5% in coronary artery bypass graft, 2.9% in caesarean section, 1.4% in cholecystectomy, 1.0% in hip prosthesis, and 0.7% in knee prosthesis [15]. Data that were collected from children seem to indicate that the risk of SSIs can be even higher, particularly in middle- and low-income countries. An international, multicentre, prospective cohort study enrolling 1159 children across 181 hospitals in 51 countries found that the SSI rate was 6.3% in children living in developed countries and 12.8% and 24.7% in those living in middle- and low-income countries, respectively [16].

This consensus document describes the use of preoperative antibiotic prophylaxis in neonates and children that are undergoing abdominal surgery and has the purpose of providing guidance to healthcare professionals who take care of children to avoid the unnecessary and dangerous use of antibiotics in these patients.

## 2. Methods

### 2.1. RAND/UCLA Appropriateness Method

This consensus document was undertaken using the Research and Development Corporation (RAND) and the University of California—Los Angeles (UCLA) appropriateness method. The RAND/UCLA method consists of the appropriateness evaluation of diagnostic and therapeutic procedures with suboptimal scientific evidence by a panel of experts [17]. According to the RAND method, a procedure is defined as “appropriate” if the expected benefits outweigh the expected negative consequences, with a wide margin that justifies it, regardless of the costs. In contrast, a procedure whose expected risks outweigh the expected benefits is considered “inappropriate”. According to the RAND definition, experts who make an appropriateness/inappropriateness judgment must consider the clinical benefits and not be influenced by economic considerations. Therefore, appropriateness is used in the evaluation of the risk/benefit ratio of a list of diagnostic, management, and therapeutic procedures [18]. For a heterogeneous topic such as surgical antimicrobial prophylaxis on which randomized controlled trials in paediatrics are lacking, the application of methods aiming to increase the homogeneity of behaviors by neonatologists, infectious diseases specialists, paediatric surgeons, and anesthetists appeared useful and appropriate. For this reason, the RAND/UCLA approach was chosen instead of GRADE methodology. Through the RAND method, the participants discussed different clinical scenarios and elaborated statements on the basis of the literature and their clinical experience. The group of experts did not consider it appropriate to combine the GRADE method with the RAND/UCLA approach because the absence of randomized studies represents a bias in defining the strength of the recommendations and in representing a consensus that is reached for real-life.

### 2.2. Recruitment of Panelists

A multidisciplinary group of experts belonging to the main Italian scientific societies dealing with anti-infective therapy of children was selected. The following Scientific Societies were involved: Italian Society of Paediatrics (SIP), Italian Society of Neonatology (SIN), Italian Society of Paediatric Infectious Diseases (SITIP), Italian Society of Infectious and Tropical Diseases (SIMIT), Italian Society of Paediatric Surgery (SICP), Italian Society of Microbiology (SIM), Italian Society of Pharmacology (SIF), Italian Society of Anaesthesia and Neonatal and Paediatric Resuscitation (SARNEPI), and Italian Society of Childhood Respiratory Diseases (SIMRI). The panel of experts was made up of 52 medical doctors with at least 5-years experience: paediatricians (*n* = 20), neonatologists (*n* = 6), infectious diseases specialists (*n* = 5), paediatric surgeons (*n* = 5), anesthetists (*n* = 8), pharmacologists (*n* = 5), and microbiologists (*n* = 3). 

### 2.3. Generation of Scenarios

Initially, a literature search was performed with a selection of documents, including randomized studies, systematic reviews of the literature, meta-analyses, and guidelines on perioperative prophylaxis for the prevention of SSI during neonatal and paediatric abdominal surgery. The literature search was carried out on the PubMed database, with a choice of articles in English that were published from 2000 until 2020. The key search terms were: “antibiotic prophylaxis” or “antimicrobial prophylaxis” AND “abdominal surgery” or “oesophagogastroduodenoscopy” or “colorectal” or “percutaneous endoscopic gastrostomy” or “gastric ulcer” or “cholangiopancreatography” or “laparoscopic” or “laparotomy” or “Tenckhoff catheter” or “small bowel” or “appendicitis” or “appendectomy” or “omphalocele” or “gastroschisis” or “ileo-colic” or “biliary tract” or “liver surgery” or “pancreas surgery” or ”liver transplantation” AND “paediatric”, “neonatal”, and “children”. Subsequently, using the Patient/Problem/Population-Intervention-Comparison/Control/Comparator-Outcome (PICO) model (i.e., defining a clinical question in terms of the specific patient problem) [17], a questionnaire was created on perioperative prophylaxis in abdominal surgery in neonatal and paediatric patients relating to different procedures, which were divided into 24 clinical scenarios. Before administration, it was tested twice with a one-week interval to a convenience sample of 4 paediatricians, 2 neonatologists, one infectious diseases specialist, one paediatric surgeon, one anesthetist, one pharmacologists, and one microbiologist. The full group of 52 participants had access to the literature review as well as approved the questionnaire. Then, 26 out of 52 experts were selected by the Scientific Societies for answering and the questionnaire was administered to 11 paediatricians, 3 neonatologists, 2 infectious diseases specialists, 3 paediatric surgeons, 4 anesthetists, 2 pharmacologists, and one microbiologist.

### 2.4. Two-Round Consensus Process

On the basis of the scenarios, the questionnaire was submitted to experts on the “REDCap” online platform. Each question included the clinical scenario and possible answers were whether or not SAP was recommended for the scenario and, in case of its recommendation, a list with all the antibiotics that were available on the EU market so that the expert could select the antibiotics that he/she considered as first choice. The selected bibliographic material was made available to all the panel members who were instructed on how to fill out the questionnaire. The experts answered the questionnaire anonymously, and their judgement was expressed on a 1–9 scale, where “1” was considered definitely inappropriate, “5” was considered uncertain, and 9 was considered definitely appropriate. Intermediate values that corresponded to different modulations of the judgement of inappropriateness (“2” and “3”), uncertainty (from “4” to “6”), and appropriateness (“7” and “8”). In evaluating each indication, each expert referred both to their own experience and clinical judgement and to the available scientific evidence. A free space was provided for any annotation or comment.

The first round of the questionnaire was blinded to the other panel members. The results of the survey were discussed in a collegial meeting to reach agreements and reduce eventual disagreements [19]. Clarifications, adaptations, and refinements of the indications and appropriateness ratings were made. A total of 16 recommendations were developed. All the 52 participants were asked to read and approve the recommendations with the availability of the full literature review in a second round during the following four weeks. 

## 3. Results

### 3.1. Principles of Preoperative Antibiotic Prophylaxis

Maximally effective antibiotic prophylaxis of SSIs should follow some simple rules. First, the prescribed drugs should be effective against the bacteria that is most likely to infect the surgical site. Regarding gastrointestinal surgery, in clean procedures, the most common pathogens are those included in the skin flora, mainly *Staphylococcus aureus* and *Staphylococcus epidermidis*, whereas in clean-contaminated procedures, most of the cases are due to enteric Gram-negative rods, mainly *Escherichia coli*, *Proteus* spp., *Klebsiella* spp., enterococci and, in some cases, anaerobes, mainly *Bacteroides* and *Clostridia* [20]. However, the prevalence of different species can vary according to the type of surgery and the moment and the site at which studies are carried out. In recent years, a significant increase in *S. aureus* detection in the USA has been reported, with a relevant portion of strains showing methicillin resistance [21]. Table 1 summarizes the main aetiologic agents according to the type of surgical procedure.

Considering the potential aetiology of SSIs, the recommended and most commonly used drugs for SSI prevention during gastrointestinal surgery are cefazolin, cefuroxime, cefoxitin, and cefotetan [14,16]. Moreover, antibiotics should be administered at an appropriate dosage and at a time which ensures the achievement of adequate serum and tissue concentrations that are effective for the killing of bacteria for the duration of the intervention. It has been established that the optimal time for the administration of preoperative doses is within 30 min before surgical incision [10,11,12]. In some cases, continuation for less than 24 h after the incision is made has been suggested [10,11,12]. Unfortunately, most of the studies have been carried out in adult patients, and very few data have been collected in children. The use of preoperative antibiotic prophylaxis in children mirrors suggestions of the guidelines that were prepared for adults. Drugs, time, and duration of administration are practically the same as those that were suggested for adults. Contrary to adults, the dosages are weight-based.

### 3.2. Gastrointestinal Endoscopy

#### 3.2.1. SCENARIO #1—Oesophagogastroduodenoscopy and Endoscopic Colorectal Procedures

All gastrointestinal endoscopy procedures can be associated with the development of infections [22,23]. The procedures can cause lesions of the intestinal wall, thus allowing the passage of bacterial flora into the bloodstream and leading to the development of bacteremia and distant infection of organs and systems. Additionally, if endoscopy involves an initially sterile site, infection can result from the use of contaminated instruments. Finally, when endoscopy requires passage through the skin, the skin incision can become the site of an infection. However, despite these premises, the use of antibiotic prophylaxis in patients that are undergoing gastrointestinal endoscopy is recommended only in selected cases. Studies have shown that the risk of infections after gastrointestinal endoscopy is generally very low. It has been reported that in adults, only oesophageal dilation [24], sclerotherapy of varices [25], instrumentation of obstructed bile ducts with endoscopic retrograde cholangiopancreatography (ERCP) [26], and percutaneous endoscopic gastrostomy (PEG) [27], are associated with a relevant risk of bacteremia, infectious endocarditis (IE), and local or distant infections.

In contrast, gastroscopy with or without biopsy, colonoscopy, colonic stent insertion, device-assisted enteroscopy, and endoscopic ultrasound (EUS) with or without fine-needle aspiration (FNA) are only exceptionally associated with infection development. This explains why the American Society for Gastrointestinal Endoscopy is extremely selective in recommending the use of prophylaxis in patients that are undergoing gastrointestinal endoscopy [28]. Practically, the routine administration of antibiotic prophylaxis solely for the prevention of bacteremia and IE is not recommended, and antibiotic administration is suggested only for patients with high-risk cardiac conditions that are suffering from gastrointestinal infections that are possibly due to enterococci. Prophylaxis is not routinely recommended for endoscopic procedures that are included EUS with or without ERCP except for cases with mediastinal or pancreatic cysts [27]. Unfortunately, no guidelines for antibiotic prophylaxis for the prevention of infections in children that are undergoing gastrointestinal endoscopic procedures are presently available.

***Recommendation 1.*** In newborn and paediatric patients that are undergoing oesophagogastroduodenoscopy and endoscopic colorectal procedures (including the execution of biopsies or the removal of polyps), oesophageal dilation with an endoscopic approach, anti-reflux plastic and therapeutic endoscopic procedures, the administration of perioperative prophylaxis is not recommended.

#### 3.2.2. SCENARIO #2—Gastroduodenal Surgery Involving the Lumen with an Application of Prosthetic Material (e.g., Percutaneous Endoscopic Gastrostomy)

PEG is commonly used to provide adequate nutritional support in patients who, despite a functional gastrointestinal system, have impaired swallowing that is assumed to last long enough to inhibit nasoenteric tube feeding [28]. Despite being generally safe, PEG placement can be associated with intra- and postoperative infectious complications that are described in up to 60% of cases. Most of them involve the peristomal site, but in some cases, systemic infections, including necrotizing fasciitis, can occur [29,30,31,32]. Several pathogens are responsible for PEG-associated SSIs. The most commonly detected are *E. coli*, *Proteus* spp., and *Klebsiella* spp. In adults, preoperative antibiotic administration to reduce PEG-associated SSIs is a well-established procedure that is strongly recommended by scientific societies. This is because several studies have shown that the rates of SSIs in patients who receive SAP could be limited to 11–17%, compared to 18–66% in those receiving placebo or no prophylaxis [31,33,34,35,36,37,38]. As single-dose and multiple-dose antibiotic regimens were found to be similarly effective [39,40], a single dose of a first- or a second-generation cephalosporin, with amoxicillin-clavulanate or ciprofloxacin as alternatives, is generally recommended. The recent emergence of methicillin-resistant *Staphylococcus aureus* (MRSA) as a PEG-site infectious pathogen has raised concerns about the use of these antibiotics. As some studies have shown significant benefits of pre-PEG MRSA screening and nasopharyngeal decolonization of MRSA in reducing the peristomal wound infection rate, this measure is recommended by some experts [41].

In children, no benefit of prophylaxis has been demonstrated [42,43,44,45,46,47,48], making the use of preoperative antibiotic prophylaxis for PEG insertion debatable. Attempts to explain why children did not benefit from this preventive measure led to the conclusion that the general characteristics of the patients that are undergoing PEG insertion may play a role in this regard. Adults are generally very prone to infections as they undergo PEG insertion mainly for malignancy or age-related clinical problems, whereas children are generally neurologic patients with a low risk of infection, and for this reason, they do not benefit from antibiotic administration. However, a deeper analysis of paediatric studies seems to indicate that antibiotic prophylaxis can also be effective in children, although with a lower efficacy than adults. In a retrospective study in which no reduction of infections after preoperative antibiotic prophylaxis was found, antibiotic use was associated with a lower fever, less frequent stoma leakage, and a shorter duration of hospitalization [14]. In a second retrospective study, without any reduction in the infection incidence in response to antibiotics, children receiving placebo had a significantly higher mean body temperature after PEG insertion, suggesting that prophylaxis could reduce the risk of bacteremia [46]. Even more interesting data suggesting the benefits from prophylaxis have been collected by a recent randomized, double-blind study in which the effect of a single IV dose of a combination of amoxicillin/clavulanic acid (50 mg/kg as amoxicillin) or 75 mg/kg ceftriaxone in penicillin-allergic patients was compared to a placebo and the rates of peristomal and systemic infections were evaluated [49]. In this study, the incidence of wound and systemic infections was lower in the treated subjects than in the untreated subjects, but differences between the groups did not reach statistical significance. However, when all surgery-related infections were considered together, the overall infection rate and the duration of hospitalization were significantly higher and longer in the placebo group than in the antibiotic group (40% vs. 13.6%, *p* = 0.04, and 4.4 ± 1.6 vs. 3.5 ± 1.05, *p* = 0.02, respectively) [49].

Despite these contradictory findings, antibiotic prophylaxis is widely used in paediatric clinical practice [50,51]. Moreover, some scientific institutions highlight that antibiotic administration can be effective in reducing SSIs in children that are undergoing PEG insertion and suggest its systematic use [52]. Drugs that are recommended for adults are the same as those used in children.

***Recommendation 2.*** In newborn and paediatric patients that are undergoing elective or emergency gastroduodenal surgery involving the lumen with the application of prosthetic material, perioperative antibiotic prophylaxis with cefazolin is recommended at a dose of 30 mg/kg (maximum dose 2 g) IV to be administered within 30 min before surgery.

#### 3.2.3. SCENARIO #3—Resection of Gastric Ulcer, Repair of Perforated Ulcer, and Revision of Gastric Emptying in Elective or Emergency Regimen

The available literature relates to adult subjects that are undergoing elective gastric surgery, in particular bariatric surgery and surgery for gastric cancer. Since these are clean-contaminated surgeries, perioperative antibiotic prophylaxis is routinely recommended, and most studies report the use of a single dose of cefazolin [53,54,55,56]. A randomized controlled trial reported the use of ampicillin-sulbactam for perioperative prophylaxis in patients that were undergoing total gastrectomy for gastric cancer, underlining the nonsuperiority of prolonging antibiotic prophylaxis beyond 24 h post-surgery in the prevention of SSI [57]. In a prospective study of obese adult patients that were undergoing Roux-en-Y gastric bypass surgery, the use of continuous infusion cefazolin reduced the rate of SSI compared to that of patients that were receiving ampicillin-sulbactam or ertapenem [58]. A single study compared the use of the combination of penicillin G and gentamicin with clindamycin and amikacin in cancer surgery, including surgery for gastric cancer: both combinations of antibiotics were shown to be safe and effective in preventing SSI when they were administered at least 30 min before the incision [59]. Studies that are conducted on large neonatal and paediatric series concerning perioperative antibiotic prophylaxis in this type of surgery are not currently available in the literature.

***Recommendation 3.*** In newborn and paediatric patients that are undergoing elective or emergency gastroduodenal surgery with the involvement of the lumen, such as resection of gastroduodenal ulcer, repair of perforated ulcer, or revision of gastric emptying, perioperative antibiotic prophylaxis with cefazolin at a dose of 30 mg/kg (maximum dose 2 g) IV is recommended to be administered within 30 min before surgery.

#### 3.2.4. SCENARIO #4—Endoscopic Retrograde Cholangiopancreatography

Regarding endoscopic retrograde cholangiopancreatography (ERCP), it has been reported that, in adult patients, this procedure is associated with a risk of cholangitis and sepsis in approximately 3% of cases and that antibiotic prophylaxis is not effective in reducing the risk of post-ERCP infections [60,61,62]. In contrast, it may increase the proportion of bacteria that are isolated from bile that are resistant to antibiotics [63,64]. Consequently, antibiotic prophylaxis is not recommended before ERCP. According to the European Society of Gastrointestinal Endoscopy [65], exceptions could be cases of anticipated incomplete biliary drainage, severely immunocompromised patients, and cholangioscopy. In these cases, the prescribed antibiotics should be active against Gram-negative bacteria and consider the local epidemiology of bacterial resistance to commonly used antimicrobials. Studies in children are few [66], and official guidelines suggesting the use of prophylaxis do not exist. A low risk of bacteremia has been reported when ERCP is performed in children without ductal obstruction. The same recommendations that were reported for adults could be used in children.

***Recommendation 4.*** In paediatric patients that are undergoing retrograde endoscopic cholangiopancreatography (ERCP), the use of perioperative prophylaxis is not recommended.

### 3.3. Abdominal Surgery with a Laparoscopic or Laparotomy Approach

#### 3.3.1. SCENARIO #5—Abdominal Surgery in an Elective Regimen, with a Laparoscopic or Laparotomy Approach, of Pyloromyotomy, Lysis of Adherent Bridle, Excision of Masses, Biopsy of the Superficial Lymphatic Structure, Hernioplasty, or Hernioraffia, with or without Means of Synthesis

Both open and laparoscopic pyloromyotomy for congenital hypertrophic pyloric stenosis are clean procedures given the absence of entry into a hollow viscus. Studies have shown that SSIs occur in no more than 1–3% of patients [67], with the greatest risk in cases of open surgery or iatrogenic perforation. Moreover, preoperative antibiotic administration has not been found to be effective in reducing the SSI incidence in adults and in a small group of children [68,69]. Considered together, these findings have led to the conclusion that antibiotic prophylaxis in children that are undergoing pyloromyotomy is not recommended. However, antibiotic prophylaxis is frequently prescribed in clinical practice. A study that was carried out in several tertiary-level children’s hospitals in the USA enrolling a total of 4206 patients showed that antibiotics were administered perioperatively in 51% of patients. Only two of 49 hospitals gave no antibiotic prophylaxis [70].

Adhesion bridle lysis surgery is classified as a clean surgery; therefore, perioperative antibiotic prophylaxis is not recommended, as in adults [71,72]. Similarly, biopsy of the superficial lymphatic structure with a laparoscopic or laparotomy approach and the removal of masses do not require SAP; however, prophylaxis with cefazolin should be considered in subjects with certain risk factors (immunosuppression, increased anesthetic risk, long-lasting intervention) [71].

Additionally, hernioplasty or hernioraffia surgery that is performed in an elective regime with a laparoscopic approach is considered a clean intervention for which the administration of perioperative antibiotic prophylaxis is not recommended. According to international guidelines for the management of inguinal hernia in the adult population in the case of laparoendoscopic repair, antibiotic prophylaxis is not recommended [73]. Similarly, systematic reviews and studies that were carried out in the adult population on the laparotomy approach did not report statistically significant differences in the rate of SSI between the group that was administered perioperative prophylaxis and the control group [74,75]. Joda also confirmed this finding in a randomized study on the paediatric population [76]. Finally, international recommendations indicate that perioperative antibiotic prophylaxis is not necessary even in the case of open correction with the use of mesh [73].

***Recommendation 5.*** In newborn and paediatric patients that are undergoing pyloromyotomy, lysis of adherent bridle, excision of masses, surgical biopsy of superficial lymphatic structure, hernioplasty, or hernioraffia in the elective regimen with laparoscopic or laparotomy approaches, the administration of perioperative antibiotic prophylaxis is not recommended.

#### 3.3.2. SCENARIO #6—Abdominal Surgery with Tenckhoff Catheter Placement with a Laparotomy Approach

The insertion of a peritoneal dialysis catheter is a procedure with an increased risk of peritonitis and exit site infections. The International Society for Peritoneal Dialysis (ISPD), in their guidelines for the insertion and management of the Tenckhoff catheter, with reference to the paediatric population, recommends the application of perioperative antibiotic prophylaxis with a first-generation cephalosporin [77]. From the analyzed studies, it emerged that the administration of cephalosporin or vancomycin as a second choice is associated with a lower rate of SSI than in patients for which an antibiotic prophylaxis was not administered [78]. Additionally, in the recommendations of the European Best Practice Guidelines, cefazolin is the antibiotic of choice for prophylaxis in this procedure [79].

***Recommendation 6.*** In abdominal surgery with Tenckhoff catheter placement with a laparotomy approach, perioperative antibiotic prophylaxis with cefazolin at a dose of 30 mg/kg (maximum dose 2 g) IV is recommended within 30 min prior to surgery.

### 3.4. SCENARIO #7- Small Bowel Surgery

Small bowel surgery includes any procedure involving incision or resection of the small intestine. Obstruction not amendable to adhesiolysis, malignancy, traumatic and nontraumatic perforation, ischaemic necrosis, inflammatory bowel disease, enterocutaneous fistula that is not amendable to closure with conservative measures, necrotizing enterocolitis with perforation, symptomatic Meckel diverticulum, or diverticular disease are the most common causes of small bowel surgery. In all of these conditions, the risk of SSIs can be as high as 20%, with the highest risk in patients with occlusion and in those with an underlying disease favoring infections [78,80,81].

Several bacterial pathogens, both Gram-negative and Gram-positive, have been associated with SSI development after small bowel surgery. However, in the absence of an obstruction, skin flora and the most common Gram-negative aerobic bacteria are the most common causes of SSIs. *E. coli*, *Enterococcus* spp., *Streptococcus* spp., *P. aeruginosa,* and *S. aureus* are the most frequently isolated strains. In contrast, both aerobic and anaerobic strains, including Clostridia, can be found in patients with occlusion [82].

All of the official guidelines recommend preoperative antibiotic prophylaxis in adults that are undergoing small bowel surgery. Cefazolin is the drug of choice, alone in cases without obstruction and in association with metronidazole when occlusion is documented. Few data have been collected in children. Among 18 patients with short bowel syndrome that were undergoing serial transverse enteroplasty, more than 50% developed infections, 6 had catheter-related bloodstream infections, 3 wound infections, and 2 urinary tract infections [83]. Lacking specific guidelines, the same recommendations that have been prepared for adults can be followed in children.

***Recommendation 7.*** In newborn and paediatric patients that are undergoing elective or emergency gastroduodenal or jejunal surgery, total or partial incision or resection of the small bowel (including packaging of enterotomy with or without enterostomy) in the absence of obstruction, perioperative prophylaxis with cefazolin with a single dose of 30 mg/kg (maximum dose 2 g) IV is recommended within 30 min before surgery. In cases of obstruction, perioperative prophylaxis with cefazolin 30 mg/kg (maximum dose 2 g) IV combined with metronidazole at a dosage of 15 mg/kg (7.5 mg/kg in infants of weight less than 1200 g; maximum dose 500 mg) IV or with IV cefotetan at a dose of 40 mg/kg (maximum dose 2 g) is recommended within 30 min before surgery.

### 3.5. SCENARIO #8—Appendectomy

Appendicitis is one of the most common causes of acute abdominal pain and the most frequent surgical emergency in paediatrics. It occurs in all age groups but it is rare in infants and significantly more common among older children and adolescents (10–19 years of age). In this age group, the annual incidence is 23.3 cases per 10,000 compared to 1–2 cases per 10,000 in the group of 0–4-year-old children [84]. However, children that are under four years of age are at the highest risk of complications [84].

According to the pathology, appendicitis is defined as uncomplicated or complicated. In uncomplicated appendicitis, which accounts for approximately 80% of the cases, an inflamed appendix is detected. Complicated cases are, on the contrary, characterized by the presence of a gangrenous or perforated appendix with or without peritonitis or abscess formation [85]. An appendectomy is the treatment of choice in complicated cases. For uncomplicated cases, surgery remains the treatment of choice, although some studies have shown that early antibiotic treatment may be associated with no need for surgical intervention in the following months [86]. Preoperative antibiotic administration to reduce the risk of SSI has been recommended and has been routinely applied for a long time in adults. The fact that SSIs occur in adults in 9–30% of uncomplicated cases and that antibiotic prophylaxis can significantly reduce the SSI incidence strongly supports this recommendation [87,88,89,90,91,92,93,94].

Only a single dose of appropriate antibiotics is suggested. Several studies have shown that multidose administration is no more effective than a single dose [91,95,96,97,98,99,100]. Recommendations need to distinguish uncomplicated and complicated appendicitis and open and laparoscopic approaches. However, it must be highlighted that preoperative antibiotic administration can be considered prophylaxis only in cases of uncomplicated appendicitis. This is because in complicated cases, antibiotic administration is generally given for several days and becomes therapy. 

The choice of antibiotics for preoperative administration has been based on the evidence that the bacteria that are detected in SSIs are anaerobes, mainly *Bacteroides fragilis*, and Gram-negative aerobes, among which, in order of importance, there are *Escherichia coli*, *Streptococcus* spp., *Staphylococcus* spp., and *Enterococcus* spp. [88,89,90,91,92,93]. Taking into account the sensitivity patterns, drug tolerability, costs, and the results of comparative studies, second-generation cephalosporins with anaerobic activity (cefoxitin or cefotetan), third-generation cephalosporins with partial aerobic activity (cefotaxime), and first-generation cephalosporins in association with metronidazole have been considered the best solutions for antibiotic prophylaxis in appendectomy. The results were generally satisfactory, showing a reduction in postoperative SSIs to less than 5% [87,88,89,90,91,92,93,94].

In children, the incidence of SSI after surgery for acute appendicitis is significantly lower than in adults. Without any antibiotic administration, postoperative infectious complications (wound infections, intraabdominal infections, or prolonged pyrexia) have been reported in only 1.2% of uncomplicated and 4.2% of gangrenous appendicitis cases [101]. Starting from these findings and the results of some studies that reported that the incidence of SSI was not substantially different in children that were receiving antibiotics or placebo [102,103], preoperative prophylaxis in paediatric patients with uncomplicated appendicitis has been questioned [95]. This conclusion was supported by a meta-analysis of 7 studies that enrolled a total of 776 patients that were aged 3 months to 15 years that found a trend towards a potential benefit of prophylaxis, but the difference compared to the placebo was not statistically significant [96]. However, as preoperative differentiation of uncomplicated from complicated cases can be difficult and antibiotic prophylaxis can reduce complication-related morbidity such as prolonged hospitalization and hospital readmission [97], antibiotic prophylaxis is presently recommended in all children with appendicitis that are undergoing surgery.

A series of studies including paediatric patients have shown that the same antibiotics that are suggested for adults are equally effective in children. Walz et al. found that ceftizoxime and cefamandole were significantly more effective than a placebo in reducing SSI and related problems such as a prolonged duration of hospitalization [96]. Lau et al. reported that the administration of cefoxitin was as effective as gentamicin/metronidazole [90], whereas O’Rourke et al. confirmed the effectiveness of intravenous cefoxitin [91]. Finally, Emil et al. found that a combination of gentamicin and clindamycin could be used with good results [98].

***Recommendation 8.*** In paediatric patients that are undergoing uncomplicated appendectomy in an emergency regimen, perioperative prophylaxis with cefazolin at a single dose of 30 mg/kg (maximum dose 2 g) IV is recommended within 30 min before intervention. In paediatric patients who undergo an emergency procedure of complicated appendectomy for a perforated appendix, perioperative prophylaxis with cefazolin with a single dose of 30 mg/kg (maximum dose 2 g) combined with metronidazole at a dosage of 15 mg/kg (7.5 mg/kg in infants weighing less than 1200 g; maximum dose 500 mg) IV or with cefotetan at a dose of 40 mg/kg (maximum dose 2 g) IV is recommended within 30 min before surgery.

### 3.6. Abdominal Wall Defect Correction Interventions

#### 3.6.1. SCENARIO #9—Omphalocele

No guidelines on perioperative antibiotic prophylaxis in neonates are currently available in the literature. The current knowledge, based on clinical practice and expert opinion, indicates that the perioperative administration of cefazolin in the case of omphalocele correction surgery is useful [99,100].

***Recommendation 9***. In neonatal patients who undergo surgery to correct omphalocele (as well as any defect of the anterior abdominal wall with a closed peritoneum), perioperative antibiotic prophylaxis with cefazolin at a dose of 30 mg/kg (maximum dose 2 g) IV is recommended within 30 min before surgery.

#### 3.6.2. SCENARIO #10—Gastroschisis

Since there are no specific guidelines on perioperative prophylaxis in the neonatal population, current clinical practice is based on expert recommendations and the experience of individual centers. In gastroschisis correction surgery, in which exposure of the peritoneum occurs, broad-spectrum antibiotic prophylaxis is generally adopted based on the combination of cefazolin and aminoglycoside [99].

***Recommendation 10.*** In neonatal patients who undergo surgery to correct anterior abdominal wall defects with an open peritoneum (e.g., gastroschisis), perioperative antibiotic prophylaxis with cefazolin with a single dose of 30 mg/kg (maximum dose 2 g) IV associated with metronidazole at a dosage of 15 mg/kg (7.5 mg/kg in neonates weighing less than 1200 g; maximum dose 500 mg) IV or with cefotetan at a dose of 40 mg/kg (maximum dose 2 g) IV is recommended within 30 min prior to surgery.

#### 3.6.3. SCENARIO #11—Oesophagus-Colon-Plastic Surgery

To date, no studies have been conducted on large paediatric and neonatal series concerning perioperative antibiotic prophylaxis in this type of intervention. In a retrospective study on the use of mechanical bowel preparation in children that were undergoing oesophageal-colon-plastic surgery, the use of ampicillin, amikacin, and metronidazole was reported from 48 h before to 7 days after surgery [104]. Being a clean-contaminated surgery with opening of the gastrointestinal tract, other groups also recommend the use of perioperative antibiotic prophylaxis with cefazolin (30–50 mg/kg, with a possible additional dose of 25 mg/kg in cases of surgery lasting more than 4 h) and metronidazole (15 mg/kg in single administration) in combination [100].

***Recommendation 11.*** In newborn and paediatric patients that are undergoing abdominal oesophageal-colon-plastic surgery, perioperative antibiotic prophylaxis with cefazolin at a single dose of 30 mg/kg (maximum dose 2 g) IV associated with metronidazole at a dosage of 15 mg/kg (7.5 mg/kg in neonates weighing less than 1200 g; maximum dose 500 mg) IV or with cefotetan at a dose of 40 mg/kg (maximum dose 2 g) IV is recommended within 30 min before surgery.

### 3.7. SCENARIO #12—Ileo-Colic Perforation

Currently, in the literature, there are no specific studies regarding perioperative prophylaxis for the prevention of SSIs, specifically in newborns and children. For adult patients, Bratzel et al. suggested a single dose of second-generation cephalosporin with activity against aerobic and anaerobic bacteria (such as cefoxitin or cefotetan) or cefazolin associated with metronidazole [105]. In the context of a high rate of resistance to cephalosporins, experts recommend a dose of ceftriaxone combined with metronidazole or ampicillin-sulbactam [105]. The combination of cefazolin, aminoglycoside, and metronidazole was recommended in a paper on antimicrobial stewardship of newborns [99]. Anandalwar et al. reported that treatment with cefazolin alone, without coverage for anaerobes for colorectal procedures was insufficient [14]. In a study on the prevention of SSIs in abdominal surgery, Danan et al. observed a higher percentage of SSIs with the administration of prophylaxis with cefotetan than with cefazolin combined with metronidazole [106].

***Recommendation 12.*** Perioperative antibiotic prophylaxis with cefazolin at a dose of 30 mg/kg (maximum dose 2 g) IV associated with metronidazole at a dosage of 15 mg/kg (7.5 mg/kg in infants weighing less than 1200 g; maximum dose 500 mg) IV or cefotetan with a single dose of 40 mg/kg (maximum dose 2 g) IV or amoxicillin-clavulanate IV at a dose, calculated on amoxicillin, of 50 mg/kg (max dose 2 g) or ampicillin + sulbactam IV at dosage, calculated on ampicillin, of 50 mg/kg (maximum dose 2 g), in the 30 min before surgery.

### 3.8. SCENARIO #13—Colorectal Procedures

Colorectal surgical procedures are complicated by SSIs in up to 40% of cases, with the highest rates in patients that are undergoing rectal procedures and those with underlying diseases favoring infection development [107]. Both aerobe and anaerobic bacteria, mainly *B. fragilis* and *E. coli*, alone or in combination, are responsible for these infections. Several studies have shown that in adults that are undergoing colorectal surgical procedures, preoperative antibiotic prophylaxis can significantly reduce the incidence of SSIs. A meta-analysis of 260 trials, including 68 different antibiotics and 43,451 participants, showed that preoperative antibiotic prophylaxis reduces the SSI rate from 39% to 13% [107]. Moreover, it was shown that although patients that were treated with single-dose antibiotics had a slightly higher risk of SSI than those that were treated with multiple-dose antibiotics, the difference could be considered compatible with appropriate benefit and harm (RR 1.30; 95% CI, 0.81 to 2.10). Finally, it was highlighted that the addition of drugs with anaerobic specificity could significantly increase the preventive efficacy of prophylaxis (RR 0.47; 95% CI, 0.31 to 0.71) and that combined oral and intravenous antibiotic prophylaxis could be more effective than the intravenous drugs alone (RR 0.56; 95% CI, 0.43 to 0.74) or the oral drugs alone (RR 0.56, 95% CI 0.40 to 0.76). Starting from these premises, the official guidelines recommend antibiotic administration in adults that are undergoing colorectal procedures [108]. However, the optimal route of antibiotic administration (oral, intravenous, or combined) is debatable because it is associated with mechanical bowel preparation. Moreover, the most effective drugs are not definitively established. Generally, a single IV dose of a second-generation cephalosporin that is effective against both aerobic and anaerobic bacteria (i.e., cefoxitin or cefotetan) or cefazolin plus metronidazole is administered within 30 min before incision [108]. Additional doses should be administered if the operation continues beyond two half-lives of the specific antibiotic [108]. Antibiotic prophylaxis should be discontinued within 24 h after the end of surgery. In geographic areas where Gram-negative rod resistance to first- and second-generation cephalosporins is high, ceftriaxone plus metronidazole, carbapenem or ampicillin-sulbactam are considered as possible alternatives [108]. In patients with beta-lactam allergies, an aminoglycoside, aztreonam, or a fluoroquinolone in association with clindamycin or metronidazole can be used. When oral drugs are used, they must cover Gram-negative rods (neomycin sulfate) and anaerobes (erythromycin or metronidazole) [108].

Unfortunately, preoperative antibiotic prophylaxis has been very poorly studied in the paediatric population [107]. This explains why significant variation exists among paediatric surgeons in the use of prophylactic antibiotics prior to colorectal procedures. However, the few studies that were carried out in children to evaluate the role of mechanical bowel preparation with or without oral antibiotics in reducing the risk of SSI after colorectal surgical procedures found that this measure had no clear benefit and may lead to worse outcomes [109,110]. A recent publication of the American Paediatric Surgical Association Outcomes and Clinical Trials Committee concluded that although studies in this regard were not available in paediatrics, intravenous preoperative antibiotic administration could be systematically recommended in children that were undergoing colorectal surgical procedures [111]. The same suggestions that are made for adults regarding the choice of the drugs and the total number of doses could be followed [111].

***Recommendation 13.*** In paediatric patients that are undergoing elective or emergency abdominal surgery with partial or total colorectal resection (including ostomy packaging), perioperative prophylaxis with cefazolin at a single dose of 30 mg/kg (maximum dose 2 g) combined with metronidazole at a dose of 15 mg/kg (7.5 mg/kg in neonates weighing less than 1200 g; maximum dose 500 mg) IV or with cefotetan IV at a dose of 40 mg/kg (maximum dose 2 g) is recommended within 30 min before surgery.

### 3.9. SCENARIO #14—Biliary Tract Procedures

Patients that are suffering from acute severe cholecystitis or acute biliary tract infection that are undergoing surgical procedures are considered patients with complicated intrabdominal infection and must receive antibiotic therapy before and after surgery [112]. In contrast, preoperative prophylaxis can be considered for patients without acute biliary tract infections or with mild to moderate cholecystitis who undergo biliary tract procedures such as cholecystectomy, exploration of the common bile duct, and choledochoenterostomy. Data that were collected in adults undergoing cholecystectomy clearly indicate that the risk of SSI development exists, although it is generally low but is higher in cases that are undergoing open procedures (1–19%) than in those that are undergoing laparoscopy (2–3%) [113,114]. Moreover, it has been shown that preoperative prophylaxis is not necessary in patients that are undergoing elective laparoscopic cholecystectomy if they can be defined as low-risk because they do not suffer from acute cholecystitis and have no history of acute cholecystitis, common bile duct calculi, jaundice, immune suppression, or prosthetic implants [112,113,114]; several studies support this conclusion. In a meta-analysis of 15 randomized controlled studies that enrolled 1494 treated patients and 1467 controls who had been treated with various antibiotics (cephalosporins, vancomycin, fluoroquinolones, metronidazole, and amoxicillin-clavulanate), SSIs were detected in 1.47% of subjects that were receiving antimicrobial prophylaxis and in 1.77% of the controls [115]. Similar findings were reported in a second meta-analysis of nine studies [116]. No need for antibiotic prophylaxis was also demonstrated in patients that were undergoing elective laparoscopic cholecystectomy with accidental or incidental gallbladder rupture and spillage of bile [105]. In contrast, antibiotic prophylaxis should be recommended for patients that are undergoing open procedures, in those undergoing laparoscopy but are at a high risk for underlying conditions or the possibility of a conversion to an open procedure [105]. However, as the identification of risk factors can be difficult, systematic administration of prophylaxis for all patients that are undergoing laparoscopic cholecystectomy could be suggested [105]. The drug of choice is cefazolin associated with metronidazole with cefotetan as an alternative. No paediatric recommendations are available. The recommendations that have been produced for adults could be used in paediatrics.

***Recommendation 14.*** In paediatric and neonatal patients that are undergoing open biliary abdominal surgery, perioperative prophylaxis with cefazolin with a single dose of 30 mg/kg (maximum dose 2 g) IV combined with metronidazole at the same time at a dosage of 15 mg/kg (7.5 mg/kg in infants weighing less than 1200 g; maximum dose 500 mg) or with cefotetan with a single dose of 40 mg/kg (maximum dose 2 g) IV is recommended 30 min before surgery.

### 3.10. Surgery on the Liver or Pancreas

#### 3.10.1. SCENARIO #15—Surgery on the Liver or Pancreas in Open or Laparoscopy

Currently, there are no paediatric studies available regarding the prevention of SSIs in abdominal surgery on the liver and pancreas, so we refer to the studies that are available for the adult population. Stack et al. concluded that broader spectrum antibiotic prophylaxis compared to cefazolin alone or cefazolin combined with metronidazole or ampicillin-sulbactam is not associated with a lower incidence of SSIs [117]. Gavazzi et al. proposed prophylaxis with antibiotics with anti-enterococcal activity in patients that were undergoing duodenum pancreatectomy with biliary stents and infected bile [118]. For biliary surgery, the Bratzler group recommends antibiotic prophylaxis with first-generation cephalosporins (such as cefazolin at a dose of 30 mg/kg), possibly repeatable in cases of surgery lasting more than four hours [99].

***Recommendation 15.*** For neonatal and paediatric patients that are undergoing abdominal surgery on the liver or pancreas in open or laparoscopy procedures, perioperative prophylaxis with cefazolin at a single dose of 30 mg/kg (maximum dose 2 g) IV is recommended within 30 min before surgery.

#### 3.10.2. SCENARIO #16—Liver Transplantation

Studies have shown that in adults that are undergoing liver transplantation, SSIs can develop in up to 37.5% of cases, leading to an increased risk of graft loss, a prolonged hospital stay, increased morbidity and mortality, and higher treatment costs [119,120,121,122]. Incisional, peritoneal, or intra-abdominal abscess infection within the first month after transplantation is mainly due to *Acinetobacter baumannii*, *Enterobacter* spp., *Escherichia coli*, *Enterococcus faecalis*, *Enterococcus faecium*, *Staphylococcus aureus,* and *Pseudomonas aeruginosa* [123,124]. This strongly suggests that antibiotic administration before surgery can be effective in reducing SSIs. Suggested antibiotic prophylaxis [125] includes piperacillin/tazobactam or cefotaxime + ampicillin. For beta-lactam-allergic patients, clindamycin or vancomycin given in combination with gentamicin, aztreonam, or a fluoroquinolone is recommended. The duration is not defined, but Bratzler et al. indicated that only one dose is usually needed and redosing is only necessary for unusually long procedures, considering the antibiotic half-life [99], whereas Abbo et al. suggested that the drugs should be given for up to 24 h after surgery [125].

Data regarding children are scarce. A retrospective study of 162 transplantations showed that in the immediate postsurgical period, a total of 235 bacterial infections were diagnosed [126]. Among them, 104 were classified as SSIs and 40 as bloodstream infections that were considered, at least in part, complications of the SSIs. A total of 30 patients (9%) died, and septic shock was the leading cause of death. Despite this, systematic perioperative and postoperative antibiotic prophylaxis was performed. Ticarcilline/clavulanic acid 75 mg/kg for 48 h was given. Antiviral, antifungal, and *Pneumocystis jirovecii* long-term prophylaxis was also administered [126]. The carrier status of multidrug-resistant bacteria and a tacrolimus level >20 ng/mL were independent risk factors for surgical site infections and the occurrence of severe sepsis or septic shock. However, no official recommendations have been produced for children. Guidelines that have been developed for adults can also be used for children with adequately modified drug dosages.

***Recommendation 16.*** In paediatric and neonatal patients that are undergoing liver transplantation, perioperative prophylaxis with amoxicillin-clavulanate at 50 mg/kg, calculated for amoxicillin (maximum dose 2 g), piperacillin + tazobactam at a dosage of 80 mg/kg, calculated for piperacillin for children between 2 and 9 months of age or of 100 mg/kg between 9 months and 40 kg, or ampicillin + sulbactam at the dosage calculated for ampicillin of 50 mg/kg (dose maximum 2 g is recommended within 30 min prior to surgery).

## 4. Discussion

The prevention of infections in children that are undergoing gastrointestinal surgical procedures has been very poorly studied. In most cases, the use of antimicrobial drugs in these conditions follows the recommendations that have been prepared for adults and does not take into account that in children, especially the youngest, susceptibility to infections is different, their gut microbiota composition and drug pharmacokinetic characteristics significantly vary, and their response to infection can be different. Specific guidelines for antibiotic prophylaxis should be prepared. On the other hand, as highlighted by the American Society of Health-System Pharmacists [99], the currently available guidelines for antibiotic prophylaxis in adults that are undergoing surgical procedures also have several limitations that require additional studies. For some procedures, the real importance of antibiotic prophylaxis is not definitively established. The role of topical antibiotic administration in limiting the risk of systemic *Staphylococcus* spp. infections or to generally reduce skin infections should be better studied. For most procedures, whether and how antibiotic administration should be continued after the conclusion of the surgery is not precisely defined for most procedures. For patients with underlying conditions causing relevant drug pharmacokinetic modifications, effective antibiotic dosages have not been established. Finally, which alternative drugs, alone or in combination, should be administered to patients who cannot use first-line drugs remains debatable. As an example, when a cefazolin in combination with metronidazole was recommended, we considered cefotetan as an alternative. However, in countries where cefotetan is not available, the combination of cefazolin and metronidazole represents the first choice. Cefoxitin could be considered instead of cefotetan for their similar characteristics, but we did not consider it as first-line therapy for the limited experience in the paediatric population. On the other hand, the RAND method did not permit to define a hierarchy of antibiotics’ administration. These limitations could be overcome only with specific paediatric clinical trials. In the meantime, this consensus document with the concurrence of experts’ opinions that were obtained with a codified consensus measurement, in association with a critical review of the published literature, allowed us to define the appropriateness of the procedures and to develop some useful recommendations for optimizing the healthcare professionals’ network related to paediatric abdominal surgery. 

Through the RAND method, in our study the participants discussed the statements that were derived from the guidelines and the agreement was reached in the recommendations for antimicrobial prophylaxis in neonates and children that were undergoing abdominal surgery. It should be noted that the participants in the project came from different clinical contexts, i.e., they were pediatricians, neonatologists, infectious diseases specialists, pediatric surgeons, anesthetists, pharmacologists, and microbiologists. For this reason, the results that were achieved demonstrate the usefulness of the RAND method for the selection of good practices and constitute the basis of an evidence-based approach. The findings that were obtained can establish the basis for educational interventions that aim to optimize the use of antibiotics. The limitations of the study included that this was an opinion-based survey to base recommendations and the agreement was reached on a collegial meeting. The lack of paediatric studies on abdominal surgery did not permit the sue of the GRADE methodology and the complexity of the topic required an on-line face-to-face meeting with all the participants. 

Considering the indispensable role of innate immune cells in host defense in surgical wounds, enhancing their function may represent a potential strategy for the prevention of SSIs [127]. Similarly, specific genetic mutations have been associated with the intestinal and skin microbiome, influencing the Shannon diversity, and *S. aureus* abundance [128,129]. Further studies should establish if and how the induction of trained immunity can best help prevent SSIs and what patient groups would most benefit as well as genetic mutations that increase the susceptibility to SSIs.

## 5. Conclusions

The present knowledge about antibiotic prophylaxis of neonates and paediatric patients that are undergoing surgical procedures should be improved. Thanks to the multidisciplinary contribution of experts belonging to the most important Italian scientific societies that take care of neonates and children, this document presents an invaluable reference tool for perioperative antibiotic prophylaxis in the paediatric and neonatal populations. Table 2 summarizes our recommendations in abdominal surgery for neonates and children. The methodological systematicity and scientific rigor that led to the drafting of this document make it usable on an international scale, with the necessary adjustments linked to the epidemiological context. However, specific studies should be planned to avoid the risk that effective recommendations that have been prepared for adults can lead to antibiotic abuse and misuse without any clinical benefit and increase the risk of adverse events and economic costs. As soon as our consensus document will be implemented by the Italian Scientific Societies, it will be interesting to analyze its clinical and economic impact in our geographical context. However, our recommendations could be generalized also in low- and middle-income countries where it has been recently highlighted the impact of simple, cost-effective, sustainable, and adaptable strategies on the reduction of SSIs morbidity risk and the associated costs [130,131,132].

## Figures and Tables

**Table 1:** Main aetiologic agents according to the type of abdominal surgical procedure.

Pathogen	Type of Surgical Procedure
*Staphyloccocus aureus*	Gastroduodenal surgery, small bowel surgery, appendectomy, biliary tract procedures
Coagulase-negative staphylococci	Gastroduodenal surgery, small bowel surgery, appendectomy, biliary tract procedures
*Escherichia coli*	Gastroduodenal surgery, small bowel surgery, appendectomy, colon surgery, biliary tract procedures
*Proteus* spp.	Gastroduodenal surgery, small bowel surgery, appendectomy, biliary tract procedures
*Klebsiella* spp.	Gastroduodenal surgery, small bowel surgery, appendectomy, biliary tract procedures
*Bacteroides* spp.	Gastroduodenal surgery, small bowel surgery, appendectomy, colon surgery
*Pseudomonas aeruginosa*	Appendectomy

Data have been extrapolated from references [1,2,10,11,12,14,15,16].

**Table 2:** Surgical antimicrobial prophylaxis in abdominal surgery for neonates and children.

Type of Surgical Procedure	Recommendation
Oesophagogastroduodenoscopy and endoscopic colorectal procedures	Not recommended.
Gastroduodenal surgery involving the lumen with an application of prosthetic mate-rial (e.g., percutaneous endoscopic gastrostomy)	Cefazolin 30 mg/kg (maximum dose 2 g) IV within 30 min before surgery.
Resection of gastric ulcer, repair of perforated ulcer and revision of gastric emptying in elective or emergency regimen	Cefazolin 30 mg/kg (maximum dose 2 g) IV within 30 min before surgery.
Endoscopic retrograde cholangiopancreatography	Not recommended.
Abdominal surgery, in an elective regimen, with a laparoscopic or laparotomy ap-proach, of pyloromyotomy, lysis of adherent bridle, excision of masses, biopsy of the superficial lymphatic structure, hernioplasty or hernioraffia with or without means of synthesis	Not recommended.
Abdominal surgery with Tenckhoff catheter placement with a laparotomy approach	Cefazolin 30 mg/kg (maximum dose 2 g) IV within 30 min before surgery.
Small bowel surgery	In the absence of obstruction, cefazolin 30 mg/kg (maximum dose 2 g) IV within 30 min before surgery. In cases of obstruction, cefazolin 30 mg/kg (maximum dose 2 g) IV combined with metronidazole 15 mg/kg (7.5 mg/kg in infants of weight less than 1200 g; maximum dose 500 mg) IV or with cefotetan 40 mg/kg (maximum dose 2 g) IV within 30 min before surgery.
Appendectomy	In uncomplicated appendectomy in an emergency regimen, cefazolin 30 mg/kg (maximum dose 2 g) IV within 30 min before surgery. In an emergency procedure of complicated appendectomy for a perforated appendix, cefazolin 30 mg/kg (maximum dose 2 g) IV combined with metronidazole 15 mg/kg (7.5 mg/kg in infants of weight less than 1200 g; maximum dose 500 mg) IV or with cefotetan 40 mg/kg (maximum dose 2 g) IV wi-thin 30 min before surgery.
Omphalocele	Cefazolin 30 mg/kg (maximum dose 2 g) IV within 30 min before surgery.
Gastroschisis	Cefazolin 30 mg/kg (maximum dose 2 g) IV combined with metronidazole 15 mg/kg (7.5 mg/kg in infants of weight less than 1200 g; maximum dose 500 mg) IV or with cefotetan 40 mg/kg (maximum dose 2 g) IV wi-thin 30 min before surgery.
Oesophagus-colon-plastic surgery	Cefazolin 30 mg/kg (maximum dose 2 g) IV combined with metronidazole 15 mg/kg (7.5 mg/kg in infants of weight less than 1200 g; maximum dose 500 mg) IV or with cefotetan 40 mg/kg (maximum dose 2 g) IV wi-thin 30 min before surgery.
Ileo-colic perforation	Cefazolin 30 mg/kg (maximum dose 2 g) IV combined with metronidazole 15 mg/kg (7.5 mg/kg in infants of weight less than 1200 g; maximum dose 500 mg) IV or with cefotetan 40 mg/kg (maximum dose 2 g) IV wi-thin 30 min before surgery or amoxicillin-clavulanate, calculated on amoxicillin, of 50 mg/kg (max dose 2 g) IV or ampicillin + sulbactam, calculated on ampicillin, of 50 mg/kg (maximum dose 2 g) IV within the 30 min before surgery.
Colorectal procedures	Cefazolin 30 mg/kg (maximum dose 2 g) IV combined with metronidazole 15 mg/kg (7.5 mg/kg in infants of weight less than 1200 g; maximum dose 500 mg) IV or with cefotetan 40 mg/kg (maximum dose 2 g) IV wi-thin 30 min before surgery.
Biliary tract procedures	Cefazolin 30 mg/kg (maximum dose 2 g) IV combined with metronidazole 15 mg/kg (7.5 mg/kg in infants of weight less than 1200 g; maximum dose 500 mg) IV or with cefotetan 40 mg/kg (maximum dose 2 g) IV wi-thin 30 min before surgery.
Surgery on the liver or pancreas in open or laparoscopy	Cefazolin 30 mg/kg (maximum dose 2 g) IV within 30 min before surgery.
Liver transplantation	Amoxicillin-clavulanate, calculated for amoxicillin, 50 mg/kg (maximum dose 2 g) IV or piperacillin + tazobactam,, calculated for piperacillin, 80 mg/kg IV for children between 2 and 9 months of age or 100 mg/kg IV between 9 months and 40 kg, or ampicillin + sulbactam, calculated for ampicillin, 50 mg/kg (maximum dose 2 g) IV within 30 min prior to surgery.
